# A Systematic Database Approach to Identify Companion Diagnostic Testing in Clinical Trials under the New In Vitro Diagnostic Medical Devices Regulation

**DOI:** 10.3390/diagnostics13122037

**Published:** 2023-06-12

**Authors:** Clara Wollenhaupt, Thomas Sudhop, Werner Knoess

**Affiliations:** 1 Department of Drug Regulatory Affairs, Pharmaceutical Institute, University of Bonn, An der Immenburg 4, 53121 Bonn, Germany; knoess@uni-bonn.de; 2 Federal Institute for Drugs and Medical Devices (BfArM), Kurt-Georg-Kiesinger-Allee 3, 53175 Bonn, Germany; thomas.sudhop@bfarm.de

**Keywords:** companion diagnostics, CDx, IVDR, personalized medicine, clinical trials, biomarker diagnostics

## Abstract

The European Union In Vitro Diagnostic Medical Devices Regulation (EU) 2017/746 (IVDR) introduces companion diagnostics (CDx) as a new legal term. CDx are applied in combination with a medicinal product to identify patient subgroups most likely to benefit from a treatment or who are at increased risk. This new regulation came into full effect on 26 May 2022 and represents the current development in personalized medicine. The implementation of IVDR and CDx is a regulatory challenge in the EU, requiring re-assessment of in vitro diagnostic medical devices (IVD) in terms of their CDx designation. To retrospectively identify IVD biomarker testing applied in clinical trials, a systematic search in the German PharmNet Clinical Trials database was developed. In total 3643 clinical trials conducted between 2004 and 2022 were identified. The results were analyzed in terms of medicinal products, biomarkers, and IVDs. Patient stratification based on biomarker testing mainly takes place in oncology-related trials, and the biomarkers most frequently tested are PD-L1 and HER2. Furthermore, there is a significant overlap between the collected data and non-European national authorities that have already implemented the CDx concept. This analysis could be indicatory of the medicinal products and corresponding IVD tests that could be CDx candidates under the IVDR.

## 1. Introduction

The field of personalized medicine has been rapidly evolving since the end of the 20th century. Knowledge of factors affecting a patient’s response to a treatment has increased, as well as the prognostic and diagnostic possibilities. In addition to the patient’s lifestyle and other factors, individual molecular characteristics play a decisive role. The key elements for the determination of molecular characteristics are adequate diagnostics for the associated biological markers. These biomarkers have a causal relation to the disease and/or to the medicinal product to be used [1]. They are predictive for the outcome of a given treatment and the patient’s prognosis. It is not only about the ability to identify the patients most likely to benefit from a treatment, but also about identifying those patients who are exposed to a greater risk by the treatment. To achieve this, in vitro diagnostic medical devices are applied to stratify patients into different subgroups based on their molecular characteristics to receive individual treatment based on their test result [2].

The implementation of these accompanying biomarker diagnostics prior to treatment with medicinal products portrays the evolution that the field of personalized medicine has undergone over the last few years. This is underlined by the new European Union In Vitro Diagnostic Medical Devices Regulation (2017/746) (IVDR), which defines these tests as companion diagnostics (CDx) [3].

The term companion diagnostics was first implemented in the European Union (EU) with the IVDR becoming effective. The IVDR repealed the old In Vitro Medical Devices Directive (98/79/EC) (IVDD) on 22 May 2022 in the European Economic Area. The IVDD did not specify on this type of accompanying biomarker diagnostics in combination with the use of medicinal products, and they were grouped together with in vitro diagnostic medical devices in general [4]. According to the definition in IVDR Article 2 (7), a CDx is a device used in combination with a medicinal product to secure its safe and effective use. It determines if a patient is eligible for a treatment with a specific medicinal product, based on measuring quantitative or qualitative biomarkers that are associated with the medical condition and the medicinal product. The aim is to identify the patient subgroups who are most likely to benefit from the treatment, as well as identifying the patients who are at increased risk of serious adverse reactions caused by the treatment with the corresponding medicinal product. Specifically excluded from this definition are devices that are used for treatment monitoring of a medicinal product [3].

Other regulatory authorities already established the term companion diagnostics, namely PMDA (Japan) in 2012, and the FDA (USA) and HCSC (Canada) in 2014 [5,6,7]. The only major difference to the EMA’s definition compared to the other regulatory authorities is the exclusion of treatment monitoring devices [5,7,8].

As a consequence of defining the new legal term companion diagnostics, new requirements were set in the EU for a subgroup of IVD devices. These new requirements for CDx devices have further implications on performance and clinical evidence. Furthermore, they require CE certification by a notified body and stricter post-market surveillance [3,9]. The CE certification is a regulatory process that ensures conformity of medical devices with EU standards for quality and safety. Consequently re-certification of the IVDs by notified bodies is necessary, as well as the involvement of the EMA or national authorities in the CDx development process [10]. The introduction of CDx devices as a new product and consequent up-regulation require an investigation into which IVDs holding market authorization fall under CDx designation. However, as a result of the non-defined scope of CDx prior to the implementation of the IVDR, companion diagnostic testing has not been consistently documented in databases of clinical trials thus far. To address this issue and to gather data on the utilization of CDx testing in clinical trials, a comprehensive database search strategy was devised. Clinical trials identified through this search strategy were examined in terms of medicinal products, biomarkers, and applied in vitro diagnostics. This analysis facilitates a strategic assessment of the potential candidates for CDx designation under IVDR, thereby aiding in the evaluation of medicinal products and corresponding IVD tests.

## 2. Materials and Methods

**Figure 1:**
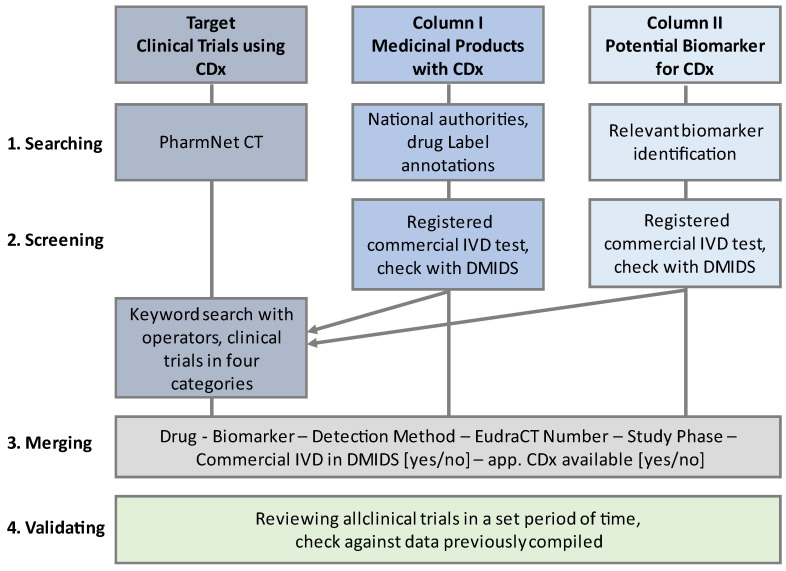
Concept of the search strategy for identifying companion diagnostics applied in clinical trials in the EU using the PharmNet CT database. The search in PharmNet CT was based on collected data of medicinal products in personalized medicine and associated biomarkers which are potentially eligible for in vitro diagnostic testing. Information on commercially available in vitro diagnostic medical devices and the applied detection method were gathered from DMIDS (German Medical Devices Information and Database System [11]). An advanced keyword search in PharmNet CT was conducted. The results from this search were documented based on the EudraCT number of the trial.

The search strategy was devised to systematically collect data on clinical trials conducted in Germany and the EU that apply CDx in their scope. It is based on extensive literature research of medicinal products used in personalized medicine and the associated biomarkers. The results were used for a systematic and widespread database research in the German PharmNet Clinical Trials database (PharmNet CT, clinical trials database of Germany’s federal and state governments). The concept of the search strategy is presented in Figure 1.

As shown in Figure 1, the fundamental literature research and data collection was based on two columns with different information included. Column I collects data on medicinal products using accompanying biomarker testing before the patient’s treatment (Column I: Medicinal Products with CDx). For Column I, the following information sources were chosen:

- List of Cleared or Approved Companion Diagnostic Devices (In Vitro and Imaging Tools), US Food and Drug Administration [12];
- Approved Pharmaceuticals With An Associated Companion Diagnostic Test, Canada’s Drug and Health Technology Agency [5];
- List of in vitro Companion Diagnostics or Medical Devices (CDx Products) Approved in Japan, Pharmaceuticals and Medical Devices Agency [7];
- The Pharmacogenomics Knowledge Base (PharmGKB), Drug Label Annotations with “Testing required” [13].

From these resources, all medicinal products and associated biomarkers were collected, as well as the trade name of the corresponding CDx and the IVD detection method. This compiled list is summarized in the Summary of Column I.

Column II collects data on potential biomarkers which can be used for companion diagnostic testing (Column II: Potential Biomarker for CDx). As in Column I, the information on biomarkers in Column II was based on different sources, as follows:

- List of Qualified Biomarkers, US Food and Drug Administration [14];
- Supplementary Tables (Table S1) on Gene–Drug Interactions, “Evaluation of Current Regulation and Guidelines of Pharmacogenomic Drug Labels: Opportunities for Improvements”, Shekhani et al. 2020 [15];
- Medicinal Products in Personalized Medicine with Marketing Authorization in Germany, Verband Forschender Arzneimittelhersteller (Germany) [16];
- Oncology drug–companion diagnostic combinations, J.T. Jørgensen 2021 [17].

Biomarkers and corresponding medicinal products were collected in the Summary of Column II. This summary was reviewed, as some biomarkers have synonyms or refer to the gene or the resulting gene product. In these cases, the biomarker was listed with alternate names in brackets affiliated.

**Figure 2:**
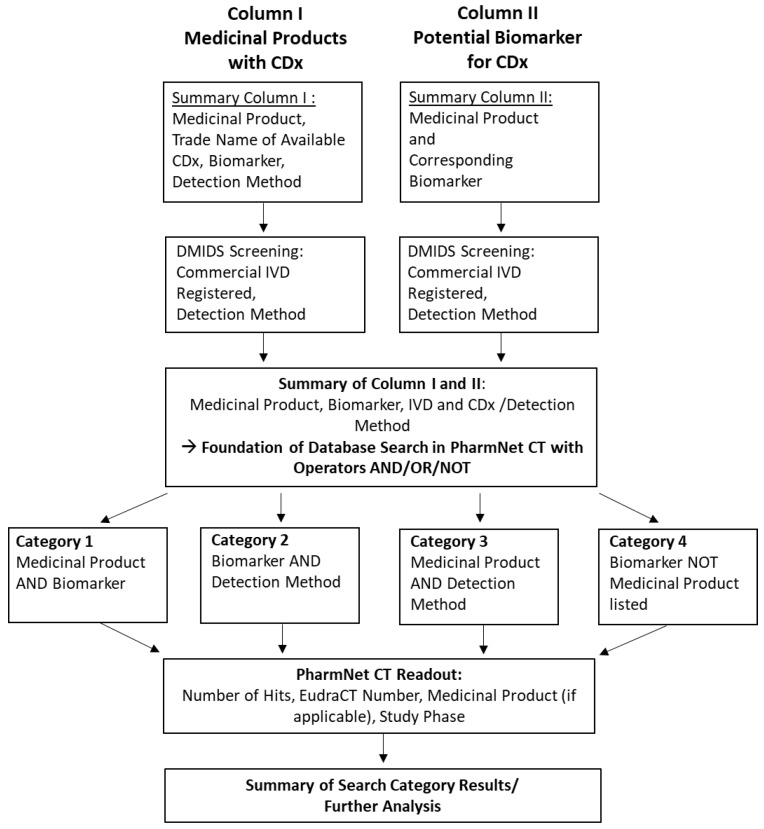
Detailed workflow. The search strategy is based on Column I (Medicinal Products with CDx) and Column II (Potential Biomarker for CDx). The information gathered in these two columns is used for a systematic keyword search in DMIDS (German Medical Devices Information and Database System [11]), integrating information on the detection method based on commercially available in vitro diagnostic medical devices. The resulting *Summary of Column I and II* is the foundation for an advanced keyword search in the PharmNet CT database, which was divided into four search categories by using different search operators.

The information acquired from both column summaries was used for a keyword search in DMIDS (German Medical Devices Information and Database System [11]). Keywords used were all biomarkers and all medicinal products identified, as well as the trade names of all IVD tests with CDx market authorization Figure 2). The screening was conducted in the section of in vitro diagnostics (“Anzeigen in In-vitro-Diagnostika”) using the search in all available text fields (“Textfelder”) of the registration form for IVD medical devices. The aim of this additional database search was to record information on the detection method of the commercially available IVD. If there was no commercial IVD registered for the corresponding biomarker, no information on the detection method was recorded (Supplementary S1). The information from both columns and the DMIDS screening was collected in the Summary of Column I and II.

The data obtained in Summary of Column I and II were the foundation for the next step (Figure 2), which included a systematic screening of keywords in the clinical trials database PharmNet CT. The database PharmNet CT only includes data on clinical trials across Europe based on applications for authorization for conducting clinical trials submitted to the German competent authorities BfArM and PEI. Nevertheless, both authorities together represent the largest within Europe, so the data generated is still likely to represent the current evolution of CDx testing applied in clinical trials.

During this search, the information on medicinal products, biomarkers, and IVD detection methods was combined using different search operators provided by PharmNet CT (Federal Institute for Drugs and Medical Devices, Cologne Office, Cologne, Germany). For detailed information on assembling the search queries in PharmNet CT, refer to the Supplementary Materials S1. Four different search categories were developed, each using different combinations of keywords or operators, as follows:

Category 1: Medicinal product AND associated biomarker;
Category 2: Biomarker AND detection method of IVD;
Category 3: Medicinal product AND detection method of associated biomarker;
Category 4: Biomarker NOT medicinal product already listed/associated (exclusion search).

The use of the different search queries mostly avoids a keyword bias by only using a combination of two keywords. In this way, a systematic exclusion of datasets can be prevented, leaving one keyword category open. With this search strategy it is also possible to find and analyze medicinal products which are still in development and have no marketing authorization. Nevertheless, as the foundation of this search is also based on the selection of biomarkers with clinical evidence, clinical trials investigating new biomarkers cannot be found with this search strategy. This creates a potential bias based on the selection of biomarkers in the preceding research in the literature. Possible errors that occurred and led to incorrect records are mainly homonyms of the keywords used or inconsistent entries in PharmNet CT. The datasets concerned were corrected accordingly or removed.

After performing the search in PharmNet CT, the resulting datasets were recorded using the EudraCT number as an identifier. Depending on the category, different readouts of the datasets were collected and recorded. The results were analyzed using the number of search results or the total count of a keyword. A validation of the process took place to evaluate the quality of the data retrieved. To achieve this, all clinical trials in 2019 and 2021 were reviewed manually and compared with the trials found by the search strategy. Overall, an error rate of 1–2% can be assumed. This refers to clinical studies that use biomarker diagnostics but could not be identified by the search strategy (Supplementary S1). In total, the number of estimated errors is relatively low compared to the total amount of data gathered in this search.

## 3. Results

### 3.1. Results Based on the Literature Research

**Figure 3:**
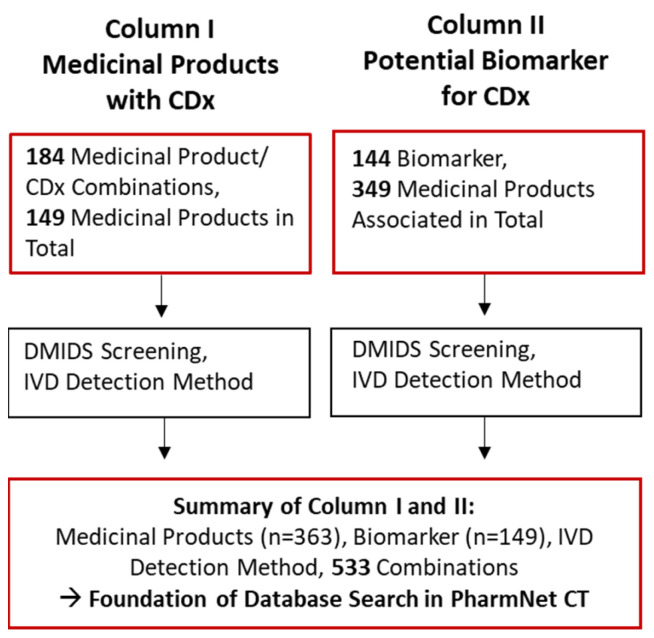
Numbers of all identified medicinal product/CDx combinations, medicinal products in total, and associated biomarkers collected from the literature research. After performing a screening in DMIDS (German Medical Devices Information and Database System) for determining the in vitro diagnostic detection method, the results are recorded and combined in the Summary of Column I and II. This summary is the foundation for the systematic database search in PharmNet CT of the accompanying biomarker testing applied in clinical trials.

A summary of the collected information based on the literature research is displayed in Figure 3. Summary of Column I and II provides the foundation of the systematic search in PharmNet CT as it includes all three elements of the CDx concept, namely the medicinal product, associated biomarker, and IVD medical device.

### 3.2. Results on Clinical Trials Identified by the Search Strategy in PharmNet CT

By combining keywords on medicinal products with an associated biomarker, it is possible is to detect which medicinal products holding marketing authorization are frequently associated with specific biomarker testing in clinical trials. The results are compared to already existing CDx designations for the FDA, PMDA, and HCSC in Table 1.

**Figure 4:**
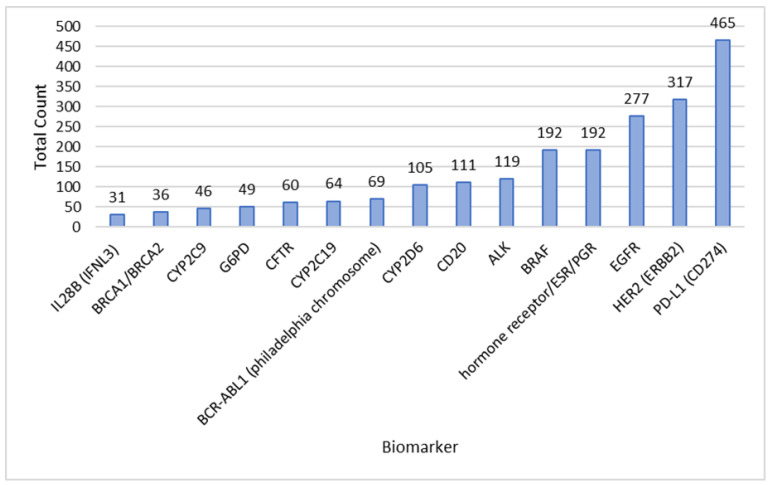
Total count of biomarkers associated with medicinal products in clinical trials acquired from database research in PharmNet CT. The cut-off is a total count of >30, results are based on the search query category “Medicinal Product AND Biomarker”. Included are clinical trials conducted in the EU from 2004–2022.

The biomarkers displayed in Table 1 can be associated with multiple medicinal products, so a total count of biomarkers was performed to investigate which biomarkers are frequently tested in the context of different clinical trials in combination with a specific medicinal product (Figure 4).

**Figure 5:**
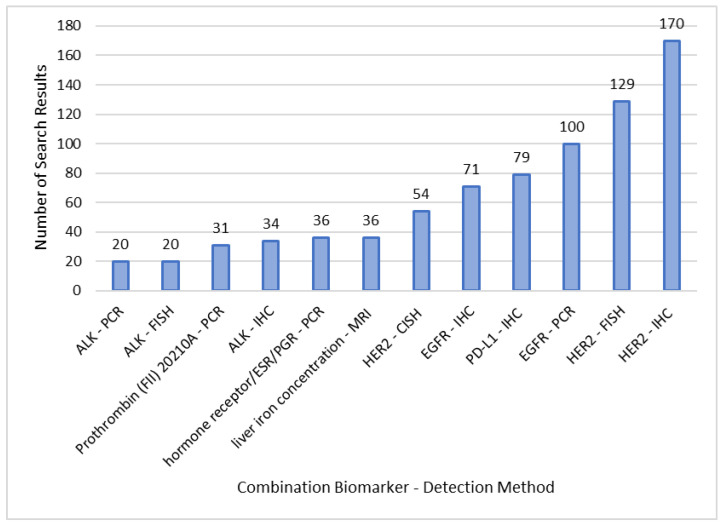
Search results of applied detection methods and associated biomarkers in clinical trials acquired from database research in PharmNet CT. The chosen cut-off is ≥20 search results, results are based on the search query category “Biomarker AND Detection Method”. Included are clinical trials conducted in the EU from 2004–2022. IHC = immunohistochemistry; FISH = fluorescence in situ hybridization; PCR = polymerase chain reaction; CISH = chromogenic in situ hybridization; MRI = magnetic resonance imaging.

Following this approach, it can be detected which IVD detection methods are preferably used for companion diagnostic testing in clinical trials (Figure 5). To achieve this, clinical trials conducting biomarker testing using a specific IVD detection method were investigated.

A further analysis was performed concerning the active substances administered in the clinical trials found by this search. Chemotherapeutic substances were excluded from this analysis, as they represent the standard of care/comparator in oncology-related clinical trials. Therefore, the accompanying biomarker testing applied in the trial is not intended for the cytostatic substance; rather, it is performed for the investigational medicinal product. For this analysis, the detected active substance and related biomarkers were compared to existing CDx designations for the FDA, PMDA, and HCSC (Table 2).

**Figure 6:**
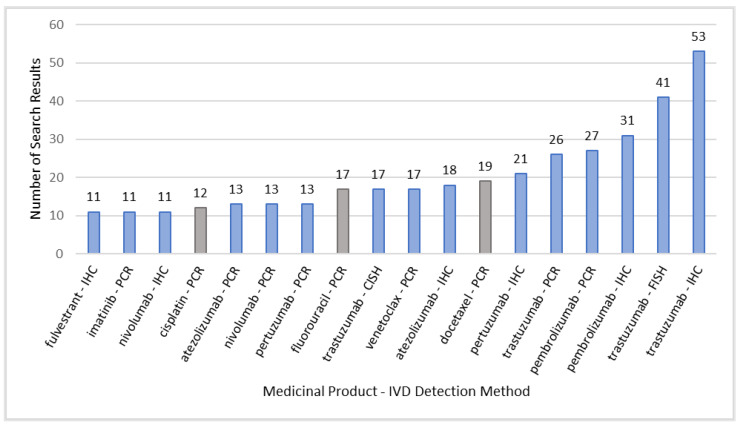
Search results of medicinal products and IVD testing methods of associated biomarkers in clinical trials. Results are determined by the number of search results in PharmNet CT, based on the search query category “Medicinal Product AND Detection Method”. The chosen cut-off is >10 search results. Chemotherapeutic substances are marked in grey as they represent the standard of care/comparator in oncology-related clinical trials. IHC = immunohistochemistry; FISH = fluorescence in situ hybridization; PCR = polymerase chain reaction; CISH = chromogenic in situ hybridization.

The results make it possible to determine which IVD detection methods are frequently applied in combination with a specific medicinal product (Figure 6). Medicinal products related to chemotherapy are marked in grey and are not part of the following analysis of IVDs in targeted therapies.

**Figure 7:**
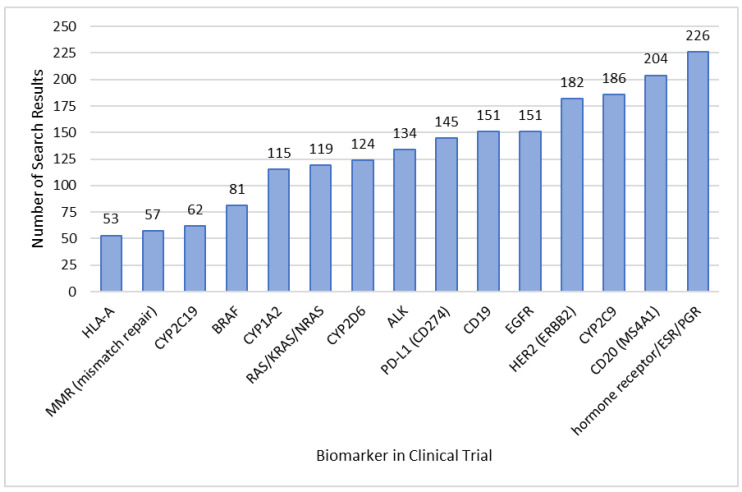
Search results of biomarkers in explorative clinical trials investigating well-established biomarkers in combination with a new associated medicinal product. The number of search results was determined by a PharmNet CT database search using datasets with the corresponding biomarker included, but excluding medicinal products already associated with the biomarker based on the literature research. The chosen cut-off is >50 search results, based on the search query category “Biomarker NOT Medicinal Product” (exclusion search).

Another search was conducted with the aim of identifying clinical trials that investigate medicinal products in a new context with a known biomarker (Figure 7). In this search category, all known biomarkers from the previous literature research were investigated. However, datasets containing an already associated medicinal product with the biomarker were excluded, using the “NOT” operator (Biomarker NOT Medicinal Product, PharmNet CT, Cologne, Germany). The investigational medicinal product of the clinical trial does not necessarily need to have a marketing authorization in the EU.

## 4. Discussion

### 4.1. Findings in Context with Applied CDx Testing

The combination of pembrolizumab and PD-L1 is the most investigated pairing of a medicinal product and biomarker, as evidenced by 171 search results (Table 1). Analyzing the surface expression of PD-L1 in tumors by partial or complete membranous staining is a very reliable tool to determine which patients can benefit from a PD-1/PD-L1 pathway inhibition, making it a prime example of companion diagnostic testing [2]. This is also underlined by the very frequent appearance of other medicinal products referring to checkpoint inhibition, for example nivolumab, atezolizumab, and durvalumab.

When comparing the top search results of medicinal products and biomarkers with approved CDx combinations (FDA, PMDA, HCSC), a strong correlation is evident (Table 1). This suggests that these medicinal product/biomarker combinations are demonstrating potential candidates for CDx testing in the EU. Looking at the clinical trials identified that 39% are investigating medicinal product/biomarker combinations that have an approved companion diagnostic IVD outside the EU, representing the extremely high demand for these specific biomarker diagnostics. Furthermore, 95% of these clinical trials are testing for associated biomarkers with suitable commercial IVDs registered in DMIDS. This shows how commonly biomarker testing is in fact performed, outlined by the high coverage of clinical trials.

Furthermore, many biomarkers associated with breast cancer can be found. This applies to the biomarker class of hormone receptors, including estrogen and progesterone receptors (ESR and PGR), as well as the growth factor receptor HER2 and the tumor suppressor proteins BRCA1/BRCA2 [27]. Overall, the primarily identified biomarkers and medicinal products are those related to oncology (Table 2). In particular, biomarkers with a long history of clinical evidence, discovered more than 20 years ago were excessively studied in different clinical contexts (for example EGFR or BRAF mutations). Consequently, these biomarkers and their associated medicinal products are frequently used as standard treatments, often serving as comparators in clinical trials involving investigational substances, explaining the significant number of trials identified involving these biomarkers and medicinal products (Figure 4). Attributable to the widespread approach, there are also biomarkers shown in this analysis related to individual drug metabolization, for example cytochrome P450 (CYP) genotyping. Nevertheless, dose adjustment and optimization of the treatment regimen are not part of the EMA’s CDx definition [3]. This excludes some biomarkers, such as CYP polymorphisms or monitoring the liver iron concentration with MRI (magnetic resonance imaging).

### 4.2. Preferred IVD Detection Methods for CDx Testing

When looking at IVD detection methods in combination with a specific biomarker, the frequency of immunohistochemistry (IHC) is particularly striking (Figure 5). Biomarkers detected by IHC are HER2 with 170 search results, PD-L1 with 79 search results, EGFR with 71 search results, and ALK with 34 search results. The US FDA approved multiple companion diagnostic tests for these biomarkers using IHC staining. Examples are the HercepTest (Genentech Inc., San Francisco, CA, USA and Roche, Basel, Switzerland) detecting HER2 for treatment with trastuzumab, PD-L1 IHC 22C3 pharmDx (Agilent Technologies Inc., Santa Clara, CA, USA) for treatment with pembrolizumab, the VENTANA ALK (D5F3) CDx assay (Ventana Medical Systems, Tucson, AZ, USA) for treatment with alectinib, and the Dako EGFR pharmDx Kit (DakoCytomation, Copenhagen, Denmark) for treatment with cetuximab [12]. Nevertheless, it must be mentioned that particularly popular biomarkers detected by IHC, such as PD-L1 and HER2, are in the spotlight of this analysis. All identified clinical trials considered, the proportion of PCR-based methods is equally high to the application of IHC. However, specific genetic alterations detected by PCR-based tests show a significantly lower prevalence, which is why they are not shown to in Figure 5.

The example of HER2 also illustrates possible differences in investigating the same biomarker with different IVD detection methods, as shown in Figure 5. Comparing IHC in situ hybridization-based methods (FISH/CISH) for the detection of HER2, it is important to mention that HER2 overexpression on the cell surface (detection with IHC) can occur without a higher copy number variation of the HER2 amplicon (detection with FISH/CISH). This makes IVDs of both detection methods inevitable, which must also be considered in CDx development and decision making [2,28].

When looking at specific active substances in combination with IVD testing, active substances can appear in combination with various IVD testing methods, which is dependent on the associated biomarker (Figure 6). For instance, the administration of trastuzumab is linked to testing for the hormone receptor status using RT-qPCR. IHC, FISH, and CISH are all used to assess the HER2 expression of tumors. Using in situ hybridization, FISH (fluorescence in situ hybridization) is preferred to CISH (chromogenic in situ hybridization) as shown in Figure 6. This appears to be a general phenomenon, as FISH is more frequently performed than CISH in all clinical trials identified by this search.

The search results also reveal a notable prevalence of checkpoint inhibitors, specifically pembrolizumab, atezolizumab, and nivolumab among the active substances identified in this analysis. All of them show high appearances in clinical trials applying IHC IVD testing. PD-L1 expression can be assessed using IHC staining or FISH to detect high-level amplification. As shown in Figure 6, only IHC staining is frequently applied in clinical trials. All approved CDx across the FDA, PMDA, and HCSC use IHC staining for the assessment of PD-L1 expression.

In general, well-established methods make up the vast majority of IVD testing in combination with a medicinal product, especially PCR-based methods (41.5%). This can also be explained by the fact that all clinical trials from 2004–2022 were included in the analysis (Figure 5 and Figure 6). New technologies, such as next generation sequencing (NGS), have been strongly emerging in the last years. This is supported by the fact that more NGS-based CDx were approved by US FDA and PMDA ongoing from 2016 [9]. NGS technology offers high accuracy, a low time of analysis, and relatively low costs. Furthermore, NGS enables the analysis of multiple genetic alterations in a single test. The proportion of NGS-based tests among already approved CDx is 39%, with an increasing tendency. It offers many advantages; one example is the FoundationOne CDx test (Foundation Medicine Inc., Cambridge, MA, USA), an NGS-based liquid biopsy platform that can detect genetic alterations in more than fifteen gene loci [29]. New established biomarkers, such as the tumor mutational burden (TMB) or microsatellite instability (MSI), are solely dependent on NGS-based detection. Biomarkers being detected by PCR or in situ hybridization-based methods can also be assessed by NGS technology [2]. In general, it can be assumed that this new technology will find similar strong application in CDx testing in the EU.

### 4.3. Emerging Biomarkers in Clinical Contexts

The findings from Figure 7 can be indicatory for emerging biomarkers in CDx development. The biomarker which is investigated most frequently in new contexts of clinical trials is the overexpression of the estrogen and progesterone receptor (226 search results, Figure 7). The high number of search results in this analysis underlines how much research in the past years was conducted in the treatment of breast cancer. The significant presence of hormone receptor status testing within this search strategy is noticeable, consistently yielding results throughout the analysis. Currently, there is no corresponding IVD with CDx approval, even though there are commercial IVD tests available determining the hormone receptor status in breast cancer (for example the OncotypeDX assay^®^, Genomic Health Inc., Redwood City, CA, USA) [30].

Another emerging biomarker in clinical contexts is MMR, with 57 search results (Figure 7). MMR refers to deficient mismatch repair and is closely connected to microsatellite instability (MSI). The US FDA approved the VENTANA MMR RxDx Panel (Ventana Medical Systems, Tucson, AZ, USA) prior to the treatment with dostarlimab, making MMR an important biomarker in CDx development [12,31].

## 5. Conclusions

The clinical trials identified were primarily oncology-related. In particular, biomarkers with a long history of clinical evidence, such as HER2 and EGFR, appear frequently, as well as checkpoint inhibitors related to the assessment of PD-L1 expression (Figure 4). Looking at the most frequent combinations of biomarkers and IVD detection methods applied in clinical trials, almost all of them have approved CDx for the FDA/PMDA/HCSC (Table 2). One exception in this analysis was testing for the hormone receptor status. Despite its very frequent usage, it has no assigned CDx approval, making it a strong possible CDx candidate in the EU.

NGS technology has been increasingly emerging in CDx development over the last years. In addition to its efficiency advantages, it also offers the possibility to investigate new biomarkers, such as MSI and TMB, making IVD testing more versatile.

Furthermore, it was determined which IVD detection methods are frequently applied in combination with a specific medicinal product, and the preferred IVD detection methods for a biomarker could be evaluated. Nonetheless, some differences can be observed between the applied IVD methods in clinical trials and the already approved CDx devices. This difference can be attributed to the different nature of clinical trials investigating a new medicinal product, standing in contrast to a medicinal product with marketing authorization and an established IVD for the detection of the biomarker. Furthermore, it must be mentioned that with the application of this search strategy no statement can be made about when the accompanying biomarker testing was performed in the clinical trial. For example, CDx testing may be performed as part of inclusion/exclusion criteria of the study or as further patient stratification into treatment subgroups.

All in all, the search strategy was shown to be broadly applicable in identifying clinical trials applying companion diagnostic biomarker testing. This systematic database approach with subsequent analysis is the first of its kind. Up to this point, only data derived from a meta-analysis of the literature were publicly available [15,32,33]. The overview of identified clinical trials provided robust data for a systematic analysis of medicinal products, biomarkers, and applied IVD testing. The results of this analysis could offer new insights in how companion diagnostic testing will be applied with the implementation of the IVDR in the EU. Regulatory agencies across the EU should observe and evaluate the experiences gained in implementing the new regulation and identify areas where optimization is needed. Special attention should be given to supporting the co-development of medicinal products and CDx. It is presumed that the selection of medicinal products eligible for CDx testing will be similar to already existing CDx combinations at the US FDA, Japanese PMDA, or Canadian HCSC. Nevertheless, one important exemption needs to be made regarding the EU definition of CDx, since devices for treatment monitoring are not included. This will also be one of the major points for the discussion of the notified bodies and the medical authorities, as there is no clear line in CDx decision making yet.

## Figures and Tables

**Table 1:** Results of the systematic database retrieval in PharmNet CT. Displayed are the medicinal product–biomarker combinations with ≥30 search results, based on the search query category “Medicinal Product AND Biomarker”. Included are clinical trials conducted in the EU from 2004–2022. Results are compared to medicinal products and associated biomarkers in alignment with IVD approved as CDx by non-European national authorities.

Medicinal Product	Biomarker	Number of Search Results	IVD Approved as CDx (National Authority)
Pembrolizumab	PD-L1	171	FDA, PMDA
Nivolumab	PD-L1	92	FDA
Rituximab	CD20	89	-
Trastuzumab	HER2	85	FDA, HCSC
Atezolizumab	PD-L1	76	FDA, PMDA
Durvalumab	PD-L1	54	-
Cetuximab	EGFR	48	FDA, HCSC
Ipilimumab	PD-L1	41	FDA *
Erlotinib	EGFR	41	FDA, HCSC, PMDA
Pembrolizumab	EGFR	39	-
Pertuzumab	HER2	33	FDA, HCSC
Ivacaftor	CFTR	32	-
Pembrolizumab	ALK	32	-

FDA = Food and Drug Administration (USA), HCSC = Health Canada/Santé Canada (Canada), PMDA = Pharmaceuticals and Medical Devices Agency (Japan). * CDx approval for a combination therapy of nivolumab and ipilimumab.

**Table 2:** Active substances acquired from an additional PharmNet CT database readout, resulting from search query category “Biomarker AND Detection Method”. Listed are only active substances of targeted therapies with related biomarkers and the approved indication. Further information on available companion diagnostic tests is added.

Active Substance	Related Biomarkers	CDx [Yes/No] (FDA/PMDA/HCSC)	Approved Indication
Trastuzumab	HER2 (ERBB2) Hormone receptor	Yes (FDA, PMDA, HCSC) No	Stomach neoplasms, breast neoplasms [18]
Pembrolizumab	ALK BRAF EGFR MMR MSI PD-L1 TMB	No No No No Yes (PMDA) Yes (FDA, PMDA) Yes (FDA)	NSCLC, melanoma, renal cell carcinoma, Hodgkin lymphoma, urologic/endometrial neoplasms, squamous cell carcinoma [19]
Atezolizumab	ALK BRAF EGFR PD-L1	No No No Yes (FDA, PMDA)	NSCLC, small cell lung carcinoma, breast- and urologic neoplasms [20]
Entrectinib	ROS1 NTRK	Yes (PMDA) Yes (PMDA)	Various cancers, NSCLC [21]
Lapatinib	HER2 (ERBB2) Hormone receptor	No No	Breast neoplasms [22]
Letrozole	Hormone receptor	No	Breast neoplasms
Bevacizumab *	VEGF *	No	Colorectal neoplasms, ovarian- and breast neoplasms, NSCLC, renal cell carcinoma [23]
Cetuximab	BRAF EGFR KRAS	Yes (FDA) Yes (FDA, HCSC) Yes (FDA, PMDA, HCSC)	Head and neck neoplasms, colorectal neoplasms [24]
Erdafitinib	FGFR2/3	Yes (FDA)	Urothelial carcinoma ** [25]

FDA = Food and Drug Administration (USA), HCSC = Health Canada/Santé Canada (Canada), PMDA = Pharmaceuticals and Medical Devices Agency (Japan). * Biomarker/medicinal product not found by previous search. Plasma VEGF is not a predictive biomarker for the treatment with bevacizumab, even though it is a VEGF-targeted therapy. Bevacizumab therapy is part of the standard of care for solid tumors [26]. ** For erdafitinib, there is no marketing authorization in the EU, although it received accelerated approval for urothelial carcinoma from the US FDA in 2019 [25].

## Data Availability

Data originating from this work is available on request from the corresponding author.

## Supplementary Materials

The following supporting information can be downloaded at: https://www.mdpi.com/article/10.3390/diagnostics13122037/s1. Supplementary S1 contains detailed information about implementing and executing the search strategy. Data on the basic literature research, the DMIDS screening, the PharmNet CT data retrieval, and the validation are available on request from the corresponding author.
