# Supplementary material for: A Systematic Database Approach to Identify Companion Diagnostic Testing in Clinical Trials under the New In Vitro Diagnostic Medical Devices Regulation
Source: Diagnostics (Basel). 2023 Jun 12;13(12):2037. doi: 10.3390/diagnostics13122037 (PMC10296913; doi:10.3390/diagnostics13122037)
Supplement: Supplementary file 1 [file diagnostics-13-02037-s001.zip › diagnostics-2383650-supplementary.pdf]

Supplementary Materials S1

S1. DMIDS Retrieval

Information on the available commercial detection method of the biomarker was based on the DMIDS screening. Access to the DMIDS database was granted by BfArM (Federal Institute for Drugs and Medicinal Products, Germany). DMIDS is the current mandatory database to be used for notification of in vitro diagnostics until EUDAMED is fully operable. It should be noted that this database only contains information on medical devices and IVDs that have been submitted by manufacturers based in Germany (or authorized representatives of manufacturers not established in a member state of the EU). Therefore, only information regarding the detection method of commercially available IVD tests was obtained from DMIDS.

Keywords used were all biomarkers and all medicinal products found in the previous data collection, as well as the trade names of IVD tests with CDx designation outside the EU. The retrieval was conducted in the section of in vitro diagnostics ("Anzeigen in In-vitro-Diagnostika") using the search in all available text fields ("Textfelder") of the registration form for IVD medical devices (Figure S1).

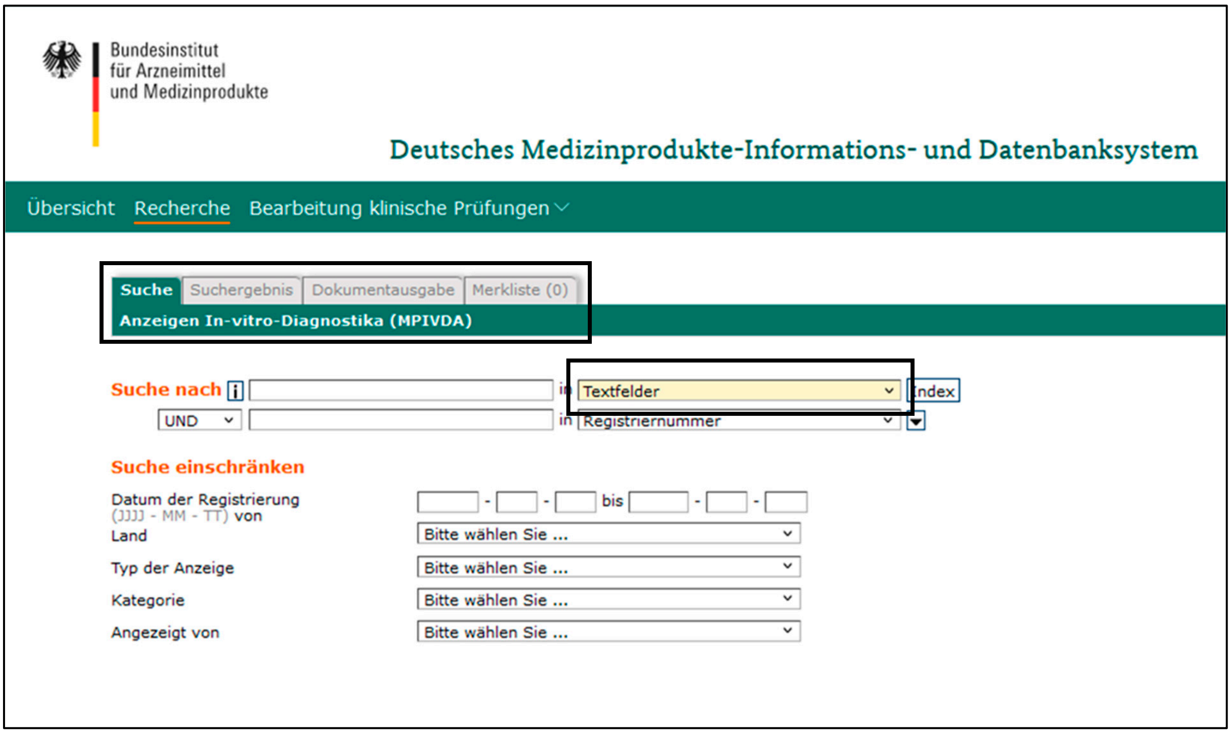

Figure S1. Search interface in DMIDS (Deutsches Medizinprodukte Informations und Datenbanksystem). Under the category "Anzeigen in In-vitro-Diagnostika" the selected keywords are entered as free-text searches in all available text fields of the data sets ("Textfelder").

The results of the database search are then displayed in the next step, showing notifications of IVDs that contain the corresponding search term (Figure S2).

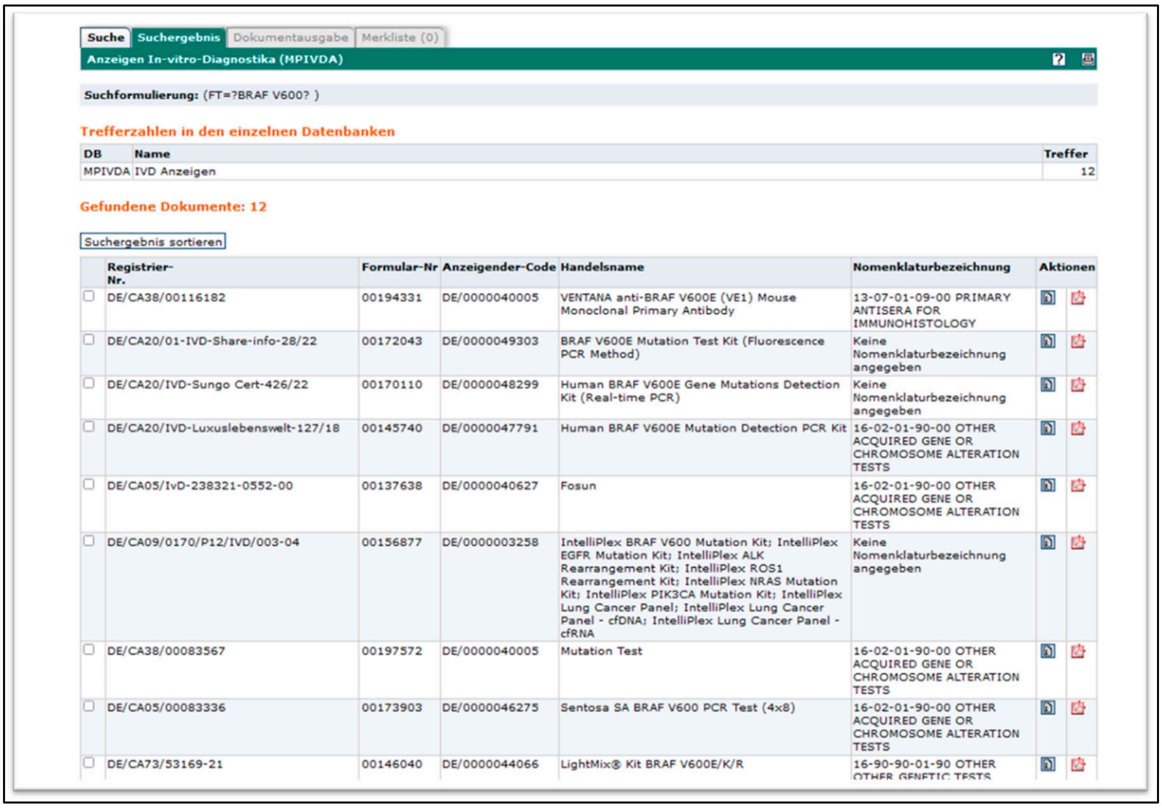

Suche | Suchergebnis | Dokumentausgabe | Merkliste (0)

Anzeigen In-vitro-Diagnostika (MPIVDA)

Suchformulierung: (FT=?BRAF V600?)

Trefferzahlen in den einzelnen Datenbanken

DB	Name	Treffer
MPIVDA	IVD Anzeigen	12

Gefundene Dokumente: 12

Suchergebnis sortieren

Registrier-Nr.	Formular-Nr	Anzeigender-Code	Handelsname	Nomenklaturbezeichnung	Aktionen
DE/CA38/00116182	00194331	DE/0000040005	VENTANA anti-BRAF V600E (VE1) Mouse Monoclonal Primary Antibody	13-07-01-09-00 PRIMARY ANTISERA FOR IMMUNOHISTOLOGY	
DE/CA20/01-IVD-Share-info-28/22	00172043	DE/0000049303	BRAF V600E Mutation Test Kit (Fluorescence PCR Method)	Keine Nomenklaturbezeichnung angegeben	
DE/CA20/IVD-Sungo Cert-426/22	00170110	DE/0000048299	Human BRAF V600E Gene Mutations Detection Kit (Real-time PCR)	Keine Nomenklaturbezeichnung angegeben	
DE/CA20/IVD-Luxuslebenswelt-127/18	00145740	DE/0000047791	Human BRAF V600E Mutation Detection PCR Kit	16-02-01-90-00 OTHER ACQUIRED GENE OR CHROMOSOME ALTERATION TESTS	
DE/CA05/IvD-238321-0552-00	00137638	DE/0000040627	Fosun	16-02-01-90-00 OTHER ACQUIRED GENE OR CHROMOSOME ALTERATION TESTS	
DE/CA09/0170/P12/IVD/003-04	00156877	DE/0000003258	IntelliPlex BRAF V600 Mutation Kit; IntelliPlex EGFR Mutation Kit; IntelliPlex ALK Rearrangement Kit; IntelliPlex ROS1 Rearrangement Kit; IntelliPlex NRAS Mutation Kit; IntelliPlex PIK3CA Mutation Kit; IntelliPlex Lung Cancer Panel; IntelliPlex Lung Cancer Panel - cfDNA; IntelliPlex Lung Cancer Panel - cfRNA	Keine Nomenklaturbezeichnung angegeben	
DE/CA38/00083567	00197572	DE/0000040005	Mutation Test	16-02-01-90-00 OTHER ACQUIRED GENE OR CHROMOSOME ALTERATION TESTS	
DE/CA05/00083336	00173903	DE/0000046275	Sentosa SA BRAF V600 PCR Test (4x8)	16-02-01-90-00 OTHER ACQUIRED GENE OR CHROMOSOME ALTERATION TESTS	
DE/CA73/53169-21	00146040	DE/0000044066	LightMix® Kit BRAF V600E/K/R	16-90-90-01-90 OTHER OTHER GENETIC TESTS	

Figure S2. Exemplary search results in DMIDS for the search term "BRAF V600". Listed are notifications of IVDs that specifically detect the BRAF V600 mutation.

Recorded information was: Is a commercial IVD for the detection of the biomarker registered [yes/no], or [yes, specific], if there was a specific mentioning of the drug in the product description of the IVD, as well as the detection method of the biomarker used by the commercial IVD. If there was no commercial IVD registered, no information of the detection method of the biomarker was recorded. This affects the keyword search in categories 2 and 3 since the keyword of the detection method is used. 30 of the 363 medicinal products could not be assigned a detection method for the associated biomarker. It is likely that rather uncommon or less frequently tested biomarkers show no results for a commercially available IVD test in DMIDS. The information from both columns and the DMIDS screening was collected in "Summary of Column I and II".

The product Information of the IVD test is worded in general terms, so the detection method of the biomarker is only basic and not detailed. Examples for these general terms are PCR (Polymerase Chain Reaction), IHC (Immunohistochemistry), FISH (Fluorescence in Situ Hybridization) or NGS (Next Generation Sequencing). Nevertheless, this basic information gathered from DMIDS showed to be more reliable than more specific terms. Examples for very specific detection methods are MLPA (Multiplex Ligation-dependent Probe Amplification), ASPE (Allele-Specific Primer Extension), or microarray-based technologies. When searching for very specific terms, little to no results are retrieved in PharmNet CT. This can be explained by the fact that in PharmNet CT only basic information on the biomarker testing is published. The advantage of the general keywords like "PCR" is also the fact that all resulting data sets of clinical trials containing "PCR" are shown, so qPCR/real-time PCR and RT-PCR are also covered by this general search term.

S2. Assembling a Search Query in PharmNet CT

The database PharmNet CT is part of PharmNet.Bund, a portal of Germany's federal and state governments, hosting information on medicinal products in Germany. The database is publicly available, however more search tools are available with an authority admission. Access to PharmNet CT database was granted by BfArM (Federal Institute for Drugs and Medicinal Products, Germany).

The available operators which can be used in PharmNet CT are "AND", "OR" and "NOT" (in German "UND", "ODER", "NICHT"). "AND" combines keywords in a search, the readout are data sets containing all keywords used. "OR" selects data sets containing at least one keyword. The last operator "NOT" is an exclusion operator, the readout are all data sets not containing the specific keyword.

In PharmNet CT it must be selected, what kind of data is being searched for. In case of medicinal products, the tab "*Medicinal product*" needs to be selected. The search window offers a drop-down menu, the category "Active substance" must be chosen (Figure S3).

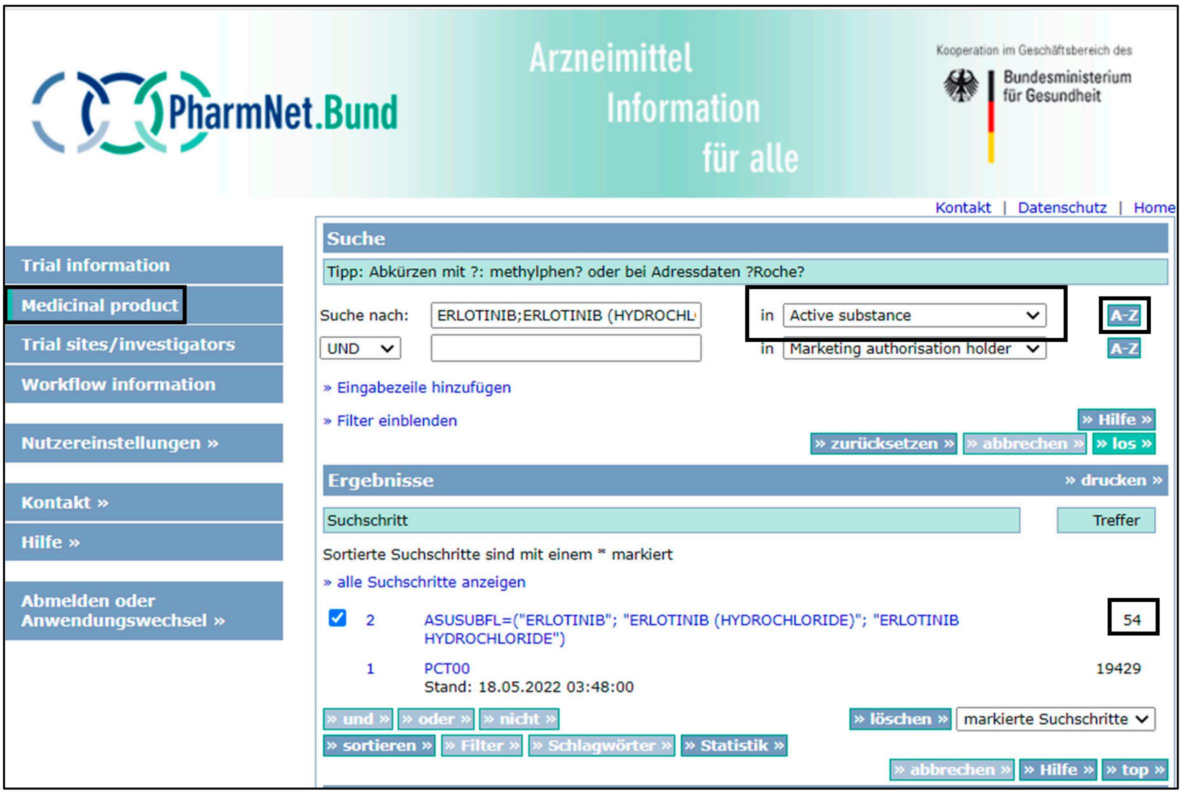

Figure S3. Searching for a specific medicinal product in PharmNet CT. Selection of the tab "Medicinal product" and "Active Substance" in the drop-down menu. Entering the INN substance name into the input field. Choice of catchwords listed at "A-Z". Number of results listed below the search window.

In a next step the search field can be used to enter the INN of the medicinal product. PharmNet CT offers a selection of catchwords including different formulations of the medicinal product based on the entry. The search process within the database is started by clicking on "go"

(German “los”). After that, the exact search properties and the number of results is shown in a window below. All data sets of clinical trials fulfilling the search criteria are also listed and can be inspected individually. The data sets can be selected for a tabular readout. The table can be generated with individual assembled columns, a readout of the EudraCT number, trial phase and the active substance (if applicable) was chosen for the following analysis. The readout format for the readout in this work was HTML. Then the data was transferred to Microsoft Excel, resulting in the different Excel sheets provided as supplementary material.

Using other keywords for the search like biomarkers or IVD detection methods, the tab “Trial information” is selected (Figure S4).

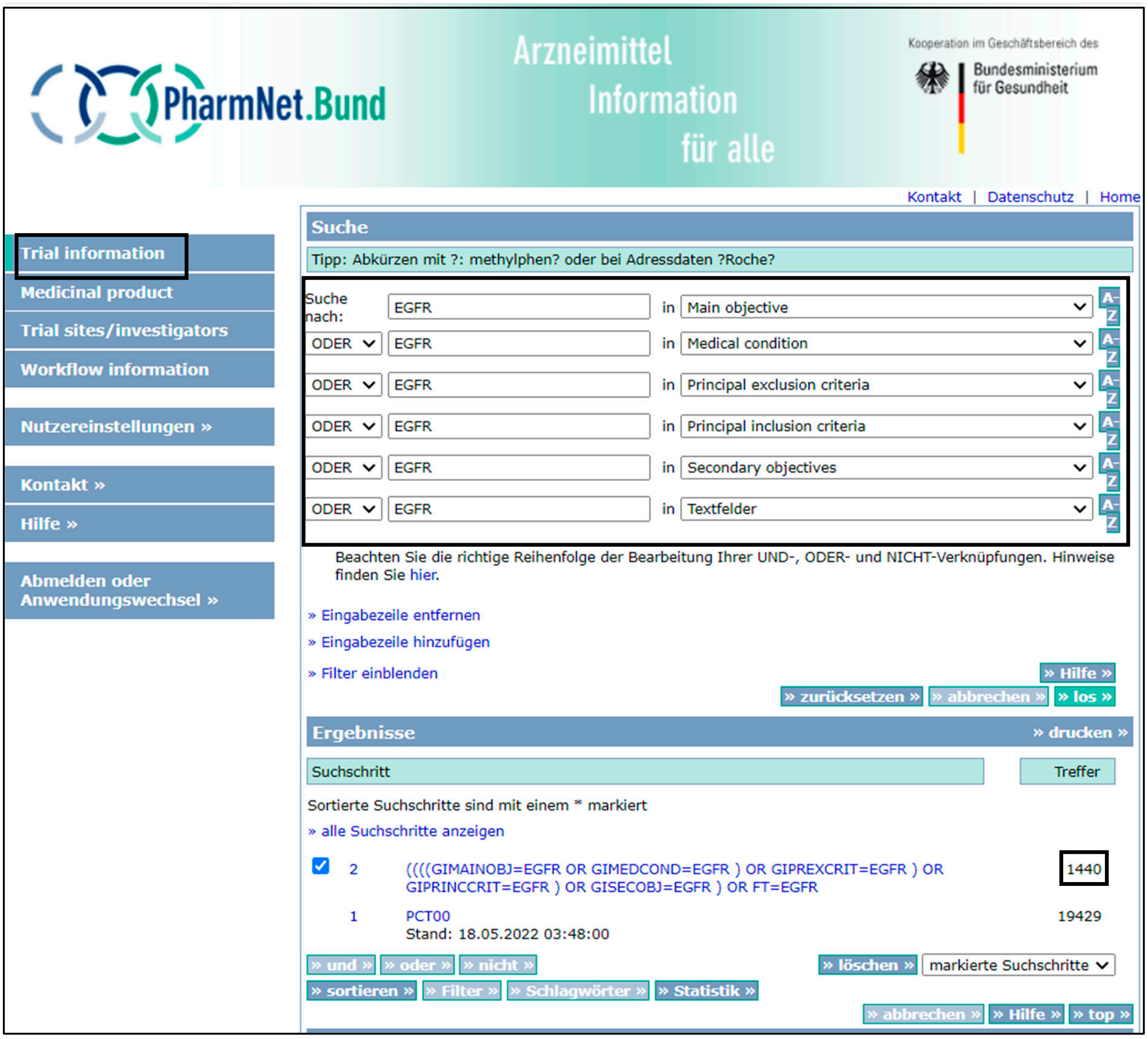

Figure S4. Searching for keywords other than medicinal products in PharmNet CT, applies to biomarkers and IVD detection methods. Selection of the tab “Trial information”. For the drop-down menu select: Main objective, medical condition, principal exclusion criteria, principal inclusion criteria, secondary objectives, and text fields (“Textfelder”). Connect the Input lines with the operator “OR” (“ODER”). The number of results is listed below the search window.

The keywords must be found in the application form, independent from where there are specifically listed. Therefore, more input lines need to be added before conducting a search in "Trial information". Six categories in the different input lines were chosen: Main objective, medical condition, principal exclusion criteria, principal inclusion criteria, secondary objectives, and text fields ("Textfelder"). An example for this type of search is shown in Figure 4. All these six input lines need to be combined with the search operator "OR", since the readout must be all data sets containing the keyword in at least one of these categories. Again, catchwords can be selected based on the entry in the input line.

For assembling a query with an operator, the resulting data sets of the search must be marked with a tick. Afterwards the required operator below can be selected by clicking on "AND", "OR" or "NOT" ("UND", "ODER", "NICHT"). The database provides the desired overlap of the data sets as a readout. In the example of Figure S5, all data sets are targeted, which contain the medicinal product "erlotinib" and in addition contain the biomarker "EGFR". The number of clinical trials containing both criteria in this example is 41. All performed steps during the search are listed and traceable. They can be selected and combined with the operators below, enabling a wide range of search possibilities.

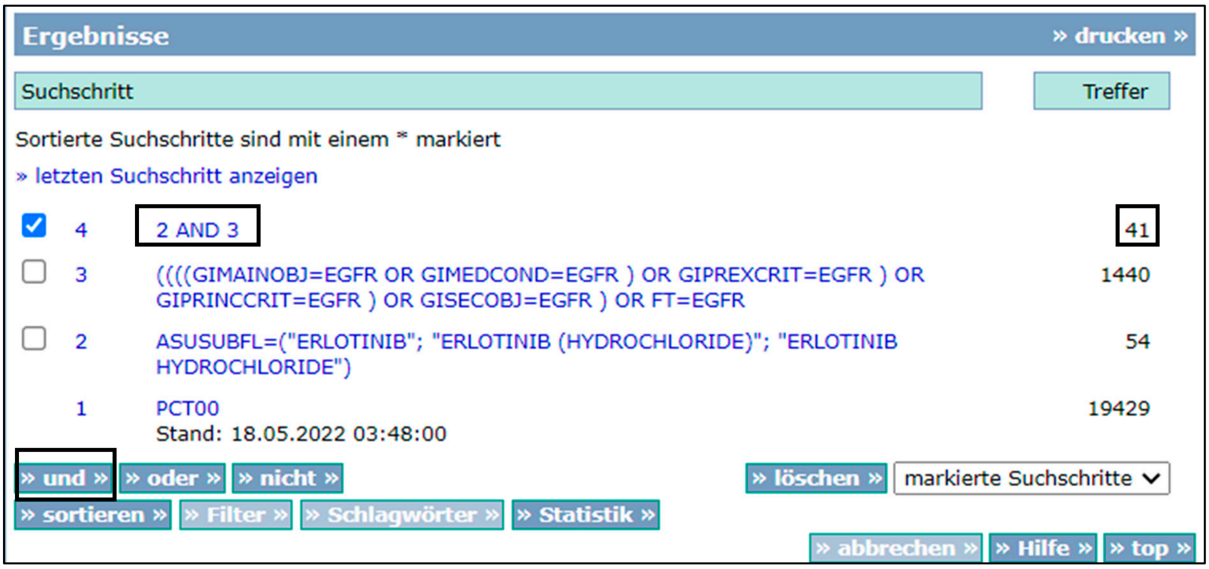

Figure S5. Assembling a search query using the operator "AND" ("UND") in PharmNet CT. Combining the data sets by selecting them with a tick mark and choosing the operator. PharmNet CT shows the subset of clinical trials containing both the medicinal product and the biomarker. Results of the query are shown in the window below on the website.

The "OR" operator was mainly used during this search to also find all data sets using alternate names of the keywords. An example is HER2 and *ERBB2*. Two separate searches for both keywords are performed, which are then connected in the next step by the operator "OR". The result of this combined "OR" query, is the basis for a subsequent query.

S3. Data Readout of the Four Different Search Categories

In Table S1, the four different search categories are summarized regarding the data readout in PharmNet CT and integration of additional data from "Summary of Column I and Column II". Depending on the search category, this information can differ. For example, in Category 2 and Category 4 an additional readout of the administered substance is possible since a medicinal product is not part of the search query. Category 2 includes the IVD detection method gathered from DMIDS, making an additional information on a DMIDS registration unnecessary.

Table S1. Displayed are the four search categories for the systematic identification of accompanying biomarker testing applied in clinical trials. The database PharmNet CT offers the use of search operators (AND, OR, NOT), resulting in the four different categories connecting different types of keywords (medicinal products, biomarkers, IVD detection methods). Depending on the category, the readout of the data sets and the added information from Summary of Column I and Column II differs.

	**Category 1**	**Category 2**	**Category 3**	**Category 4**
**Type of Search Query**	Medicinal product AND biomarker	Biomarker AND detection method	Medicinal product AND detection method	Biomarker, NOT medicinal product listed, exclusion search
**Data Readout**	Number of search results, EudraCT number, study phase	Number of Search results, EudraCT number, study phase **Additional:** Administered Substance	Number of Search results, EudraCT number, study phase	Number of Search results, EudraCT number, study phase **Additional:** Administered Substance
**Integration of Additional Data from *Summary of Column I and Column II***	Commercial IVD in DMIDS, approved CDx available, Origin of the MP [Column I or II]	Approved CDx available	Commercial IVD in DMIDS, approved CDx available, Origin of the MP [Column I or II]	Commercial IVD in DMIDS, approved CDx available

MP = Medicinal product

S4. Recording and Processing of the Identified Clinical Trials

Microsoft Excel is a suitable option for recording and analyzing the clinical trials gathered from this search. Each search category received a district sheet in since the readout can be different. Examples are Category 2 and Category 4; both have the active substances administered in the trials as an additional result recorded. The results are subdivided in the four search categories and an overview of all clinical trials found, distinguishable by the EudraCT number . Further information recorded in this general overview of the clinical trials is the study phase and the origin of the search category.

The filtering options offer various possibilities of selecting specific information in the different rows of the table based on medicinal products, biomarkers, IVD detection methods and CDx designation. Moreover, the different analysis functions can be helpful to analyze this great quantity of data, like [COUNTIF] or [XLOOKUP]. There are many ways to analyze this data, the analysis only makes use of a small proportion. It is recommended to have a close look into the raw data, as specific and customized questions of interest can be answered.

In Table S2 the numbers and results from the different categories are summarized. As shown by the number of rows in Microsoft Excel , the amount of data gathered in this search is enormous. Nevertheless, it is important to mention that one specific clinical trial can be found more than once using different keyword combinations in one category. To finally receive the number of trials found, the "*remove duplicates*" feature was used. It narrows down the number of results by half, underlining the importance of this step.

Table S2. Summarizing the number of results from the categories 1-4 based on the search category of finding clinical trials using CDx. Results displayed are the number of rows in Microsoft Excel, the number of search results/total hits in one category and the resulting number of clinical trials (without duplicates).

	Calculated Number of Queries	Resulting Rows in Excel	Number of Search Results	Number of Trials Without Duplicates
**Category 1**	1,045	2,664	2,398	1,497
**Category 2**	283	4,514	1,209	641
**Category 3**	1302	1,674	944	458
**Category 4**	438	7,959	3,024	2,309
**Total**	3,068	16,811	7,575	3,643

S5. Analysis Tools

In general, there were two main options to analyze and quantify the data from the categories gathered in Microsoft Excel.

Number of Search Results

The first possibility was to analyze the number of search results, which is always the outcome of a specific combination of keywords/search query. For instance, the number of search results emerging from the combination of "nilotinib" AND "BCR-ABL1 (Philadelphia chromosome)". Based on the structure of the Microsoft Excel sheet, the number of wanted search results can easily be selected by the filtering function. In this way, an overview on the most common keyword combinations can be gained by evaluating the number of search results and choosing an appropriate cut-off.

Total Count of a Keyword

The second option was using the total count of a specific keyword in a Microsoft Excel sheet. This is to evaluate how often a biomarker is used in clinical trials, since one biomarker can be associated with multiple medicinal products. Depending on the category, the Microsoft Excel sheet is designed in the way that each clinical trial has one row (Category 1 and Category 3), each associated with the keyword. This fact can be used to count the rows containing this keyword. To perform this count, an auxiliary table needs to be prepared on a different sheet, making use of the Microsoft Excel function [=COUNTIF]. This function reliably counts a search criterion in another matrix.

S6. Validation of the Search Strategy

For the validation of the search strategy all clinical trials in a set period of time were reviewed manually and compared to the clinical trials found by the search strategy in PharmNet CT. Assessed were the years 2019 and 2021. These years were chosen as they represent timeframes before and during the COVID-19 pandemic. It is estimated that not only did the total number of clinical trials change during the pandemic, but also the type of trials and the substances administered. The clinical trials in this timeframe were selected by the national competent authority decision date on the application of conducting a clinical trial. In 2019, BfArM and PEI decided on 951 clinical trial applications. In contrast, in 2021, there were only 722. The recorded clinical trials in these two years are reviewed manually and compared to the results of the search strategy developed. There are three different types of clinical trials recorded:

- Clinical trial was identified by the search strategy, manual review confirms applied biomarker testing; Correct.
- Clinical trial was not identified by search strategy, manual review confirms that no additional biomarker testing was applied in the trial; Correct.
- Clinical trial was not identified by the search strategy, manual review confirms applied biomarker testing prior to treatment/biomarker-based patient stratification; Incorrect.

In 2019, 15 of 951 clinical trials used accompanying biomarker testing that was not captured by the search strategy. 8 of 772 clinical trials were not recorded in 2021. This corresponds to an estimated error of 1-2 % of clinical trials that could not be detected by the search strategy. The entire validation can be viewed upon request. Overall, the search strategy showed to generate consistent results in identifying appropriate clinical trials. One limitation that became apparent is due to the selection of biomarkers for the systematic database research. In addition, some clinical trials include biomarker sub-studies which are not defined more precisely in PharmNet CT and therefore cannot be detected by the search strategy.
